# Strategies to Increase Peer Support Specialists’ Capacity to Use Digital Technology in the Era of COVID-19: Pre-Post Study

**DOI:** 10.2196/20429

**Published:** 2020-07-23

**Authors:** Karen L Fortuna, Amanda L Myers, Danielle Walsh, Robert Walker, George Mois, Jessica M Brooks

**Affiliations:** 1 Department of Psychiatry Geisel School of Medicine Dartmouth College Concord, NH United States; 2 Department of Public Health Rivier University Nashua, NH United States; 3 Department of Psychology Framingham State University Framingham, MA United States; 4 Massachusetts Department of Mental Health Boston, MA United States; 5 School of Social Work University of Georgia Athens, GA United States; 6 School of Nursing Columbia University New York, NY United States

**Keywords:** COVID-19, peer support, telemental health, mental health, training

## Abstract

**Background:**

Prior to the outbreak of coronavirus disease (COVID-19), telemental health to support mental health services was primarily designed for individuals with professional clinical degrees, such as psychologists, psychiatrists, registered nurses, and licensed clinical social workers. For the first the time in history, peer support specialists are offering Medicaid-reimbursable telemental health services during the COVID-19 crisis; however, little effort has been made to train peer support specialists on telehealth practice and delivery.

**Objective:**

The aim of this study was to explore the impact of the Digital Peer Support Certification on peer support specialists’ capacity to use digital peer support technology.

**Methods:**

The Digital Peer Support Certification was co-produced with peer support specialists and included an education and simulation training session, synchronous and asynchronous support services, and audit and feedback. Participants included 9 certified peer support specialists between the ages of 25 and 54 years (mean 39 years) who were employed as peer support specialists for 1 to 11 years (mean 4.25 years) and had access to a work-funded smartphone device and data plan. A pre-post design was implemented to examine the impact of the Digital Peer Support Certification on peer support specialists’ capacity to use technology over a 3-month timeframe. Data were collected at baseline, 1 month, 2 months, and 3 months.

**Results:**

Overall, an upward trend in peer support specialists’ capacity to offer digital peer support occurred during the 3-month certification period.

**Conclusions:**

The Digital Peer Support Certification shows promising evidence of increasing the capacity of peer support specialists to use specific digital peer support technology features. Our findings also highlighted that this capacity was less likely to increase with training alone and that a combinational knowledge translation approach that includes both training and management will be more successful.

## Introduction

Digital peer support has potential to expand the reach of peer support services, improve the impact of peer support without the need for in-person sessions, and increase engagement among mental health service users [1-3]. Digital peer support is defined as live or automated peer support services delivered through technology mediums [4]. Peer support services are recovery and wellness support services provided by an individual with a lived experience of recovery from a mental health condition [5]. Most existing telemental health training is designed for individuals who have professional clinical degrees and licensures, such as psychiatrists, psychologists, registered nurses, and social workers [6,7]. These training sessions are short in duration [6], build on already existing skill sets, and focus on rapid attainment of skills and concepts [6]. Digital peer support is quickly expanding worldwide in the wake of the COVID-19 pandemic [3]; therefore, telemental health training developed for peer support specialists is currently needed.

Academic training programs for clinicians (eg, psychiatrists, psychologists, registered nurses, and licensed clinical social workers) frequently address methods and best practices for implementing telemental health services [7]. Within these traditional clinical roles, clinicians are encouraged to explore telemental health services through formal education standards and licensure requirements, continuing education credits, national training centers, professional associations, incentives for clinicians to use telehealth modalities [8], and reimbursement for telemental health services in private and public health systems [9]. Peer support specialists are increasingly reporting the desire and need to use technology to deliver peer support [10]. As peer telemental health is now reimbursable by Medicaid during the COVID-19 emergency crisis, standardized training on digital peer support services is greatly needed.

Using the framework for an Academic-Peer Partnership [11], we developed the Digital Peer Support Certification, which is designed specifically for peer support specialists (both Medicaid-billable peer specialists in traditional clinical services and peer specialists working for peer-run organizations) who deliver peer support via technology mediums in any country worldwide. This study examined the extent to which implementation of the Digital Peer Support Certification over three consecutive months impacted peer support specialists’ capacity to use a digital peer support smartphone app and care management dashboard, PeerTECH [1-3]. 

## Methods

### Study Design and Participants

A pre-post design was used to examine the 3-month Digital Peer Support Certification program offered through a community mental health center. Data were collected at baseline, 1 month, 2 months, and 3 months. This study was conducted between November 2019 and April 2020 in a community mental health center in an urban setting. The Dartmouth College institutional review board approved this study.

The participants included 9 certified peer support specialists between the ages of 25 to 54 years (mean 39). All the participants were trained and accredited as certified peer support specialists by the state of Massachusetts and were all employed for a mean of 4.25 years (range 1 to 11 years). All peer specialists personally owned or had access to a personal smartphone. 

### Digital Peer Support Certification

The 3-month Digital Peer Support Certification was co-designed with academic partners and peer support specialists using the Academic-Peer Partnership [11]. In an earlier quantitative study (under review), our co-production team conducted an online survey with 267 peer support specialists to identify factors that can either prevent or enable digital technology engagement. Based on our findings, we co-designed specific digital peer support training content to meet the specialists’ needs. The Digital Peer Support Certification includes training on digital communication skills; technology literacy (ie, important digital terms such as PEERbots and digital phenotyping); technology usage skills with the PeerTECH system (eg, downloading apps, sending SMS text messages, entering goals, saving information, completing repeated surveys such as ecological momentary assessments on a smartphone app, increasing the volume on a smartphone, watching videos in the library, and offering digital peer support services); available digital peer support technologies; organizational policies and compliance issues; separating work and personal life; digital crisis intervention; and privacy and confidentiality. The Digital Peer Support Certification includes an education and simulation training session, synchronous and asynchronous support services, and audit and feedback. To ease uptake, the format, structure, and vocabulary were designed to be aligned with national peer support specialist practice standards [12]. Next, we will delineate each component of the certification program.

#### Education and Simulation Training Session

The education and simulated training session lasted 16 hours over two consecutive days and was led by the principal investigator, KLF. Facilitated interactive group discussions were paired with a printed standardized workbook. A standardized workbook was provided to all peer support specialists. All standardized workbook text was written at a fourth grade level and incorporated recovery principles consistent with peer support specialist practice standards [11,13]. The training was consistent with person-first language, involved sharing lived experiences of using technology in a group environment, and included simulation-based training on the PeerTECH smartphone app and the PeerTECH dashboard on a desktop computer. To promote learning of new knowledge and mastery of skills, reinforcement, summation, and teach-back techniques were incorporated into the education and simulation training session.

#### Audit and Feedback

As peer support practice standards are based on experiential learning and sharing of experiences [12], experiential learning was encouraged and an audit and feedback process was incorporated into the second phase of the Digital Peer Support Certification*.* After the two-day training session, the peer support specialists applied their newly obtained technology skills for 1 month as part of PeerTECH, a 12-week digital peer support program that incorporates a smartphone app for service users and a care management dashboard to deliver peer support to service users via a smartphone app [1]. Audit and feedback is a quality improvement management tool that incorporates a summary of performance over a specific time period designed to provide constructive feedback to people so they can modify their performance [14-16]. Audit and feedback is used in all health care settings and most commonly involves clinical health professionals rather than peer support specialists [14-16].

The audit and feedback criteria were developed by two authors KLF and RW a priori. These criteria included capacity to complete peer support specialists’ technology-based PeerTECH tasks, including signing in to the dashboard with a username and password; writing an SMS text message in the dashboard and sending it to the smartphone app; and assisting service users in completing technology-based PeerTECH tasks, including entering goals on the smartphone app, signing in to the smartphone app with a username and password, completing surveys on the app, and sending SMS text messages. The audit and feedback process was performed in a group setting at 1 month during a 1.5-hour meeting and individually at 2 months with each peer support specialist via telephone and email; feedback sessions were also offered upon request. However, no additional feedback sessions were requested. The audit and feedback sessions aimed to promote digital peer support technology capacity using positive behavioral approaches [17,18]. We adopted a nonaversive behavioral approach to working with peer support specialists during the feedback sessions [19]. Nonaversive behavioral support focuses on affirmation of practices designed to educate and promote additional positive changes [20].

The principal investigator met with all peer support specialists in a group setting at baseline and after 1 month, then contacted the specialists individually at 2 months via telephone or email. Prior to the 1.5-hour group meeting at 1 month and the 15-minute individual meeting at 2 months, the principal investigator completed a technology audit and audio observations through audio recordings of PeerTECH sessions. Upon completion of both audits, descriptive statistics were calculated and prepared for the feedback meetings with the peer support specialists.

#### Synchronous and Asynchronous Support

**Figure 1:**
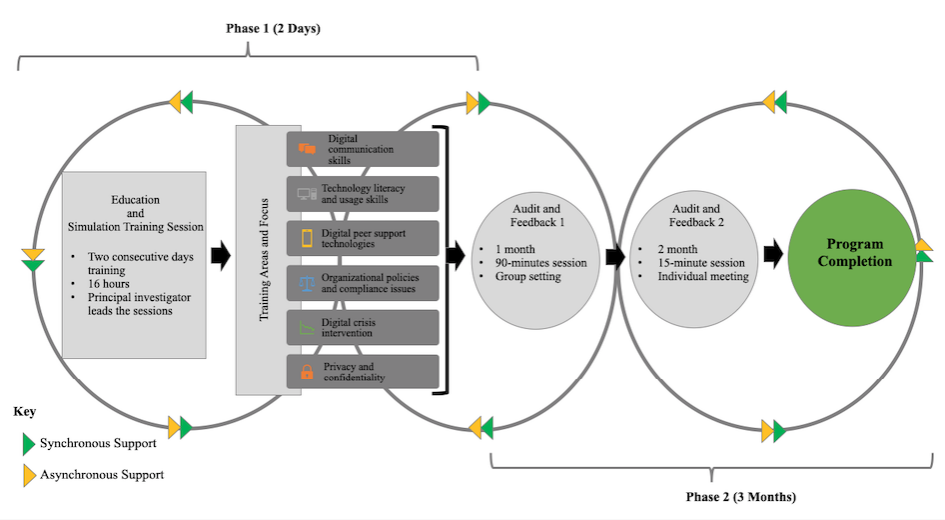
Digital Peer Support Certification Process.

Synchronous and asynchronous support were provided as needed. As such, the principal investigator and a research assistant offered telephone support (synchronous) and email support (asynchronous) from Monday to Friday between the hours of 9 AM and 5 PM. The components of the Digital Peer Support Certification are summarized in Figure 1. 

### Capacity to Use Digital Peer Support Technology

Capacity to use digital peer support was defined as the peer support specialists’ ability to use the PeerTECH system (ie, smartphone app and dashboard) through an in-person task analysis and a real-world task analysis. Task analysis is a user-centered design approach that is implemented to assess whether an individual can complete a task via a technology medium [21]. The tasks were defined based on tasks users are required to perform to operate the PeerTECH system, including signing in to the dashboard with a username and password; writing a text message in the dashboard and sending it to the smartphone app; and assisting service users in completing technology-based PeerTECH tasks, including entering goals on the smartphone app, signing in to the smartphone app with a username and password, completing surveys on the app, and sending SMS text messages. Real-world task analysis included SMS text message exchanges, entering service user goals, completion of surveys by service users, and frequency of contacting the help desk. Peer support specialists were required to send 2 text messages each week to service users and were also instructed to include at least one goal in the smartphone app.

### Data Analysis

Data from the PeerTECH system were imported into SPSS [22] (IBM Corporation) for analysis. The mean adherence from audit data from month 0 to month 1 was calculated to represent the peer support specialists’ capacity at the beginning of the certification process. The midpoint included month 1 to month 2. The mean capacity audit data from month 2 to month 3 were calculated to represent the end of the certification process for the capacity comparisons. To explore changes in the capacity to use the technology, data were calculated for SMS text message exchanges, entering service user goals, surveys completed by service users, and frequency of contacting the help desk.

## Results

### Beginning of Digital Peer Support Certification (Month 0 to Month 1)

Between baseline and **1** month, 27 service users enrolled in the study. The principal investigator downloaded the app on the service users’ smartphones or borrowed smartphone devices. Of the 27 service users, 7 (26%) borrowed a smartphone during the duration of the study.

Of the 9 peer support specialists, 3 (33%) needed password assistance a total of 4 times (ie, the peer support specialists forgot their passwords). A password reset was required for 1/9 peer support specialists (11%). No service users contacted the help desk due to forgotten passwords during this time. However, 1/27 service users (4%) required another download of the PeerTECH app. A summary of the baseline results for goals entered, surveys completed by service users, and SMS text messages sent is detailed in Table 1.

**Table 1:** Changes in peer support specialists’ capacity to use digital peer support technology from baseline to the midpoint and end of the Digital Peer Support Certification.

Capacity	Baseline (1 month)	Midpoint (2 months)	Change (%)	End of Digital Peer Support Certification program (3 months)	Change (%)
Surveys completed^a^	0	202	Infinity	397	96.5
Texts sent by peer specialists	2	19	850	89	368.4
Texts sent by service users	5	42	740	67	59.5
Goals entered by peer specialists	0	10	Infinity	16	60

^a^Service users were prompted to complete one 3-item survey on a smartphone each day for 90 days.

### Midpoint (Month 1 to Month 2)

The mean capacity from audit data for month 1 to month 2 was calculated to represent the peer support specialists’ midpoint capacity. During a 4-hour group meeting with the principal investigator, peer support specialists and their respective supervisors met to discuss PeerTECH. Between baseline and midpoint, the same 27 service users were enrolled in the study.

Between baseline and midpoint, 1/9 peer support specialists (11%) needed password assistance a total of one time (ie, they forgot their password). None of the peer support specialists required a password reset between baseline and midpoint. Service users did not contact the help desk due to forgotten passwords during this time. A summary of the midpoint results for goals entered, surveys completed by the service users, and SMS text messages sent is detailed in Table 1.

### End of Digital Peer Support Certification (Month 2 to Month 3)

The mean capacity from audit data for month 2 to month 3 was calculated to represent the midpoint capacity. The principal investigator met with peer support specialists by telephone individually, audited their work, and sent emails in PeerTECH with information related to their work. Between midpoint and end of the Digital Peer Support Certification, 1/9 peer support specialists (11%) needed password assistance a total of 1 times (ie, they forgot their password). None of the peer support specialists required a password reset between the midpoint and end of the Digital Peer Support Certification. Service users did not contact the help desk for forgotten passwords for service users during this time. Table 1 presents information on the changes in the peer support specialists’ capacity to use digital peer support technology over three months.

## Discussion

### Principal Findings

This study examined the extent to which an education and simulation training session, synchronous and asynchronous technology support services, and audit and feedback over three months impacted peer support specialists’ capacity to use digital peer support technology. The peer support specialists’ capacity was less likely to change with training alone (ie, education paired with simulation-based training); this indicates that a combinational knowledge translation approach that includes training *and* management may be more likely to improve capacity. As the need for digital mental health services has expanded due to stay-at-home measures related to the COVID-19 pandemic, peer support specialists may play a significant role in digitally supporting the needs of people by providing support services to augment traditional mental health treatment.

The combination of training and management approaches is an effective knowledge translation intervention to increase peer support specialists’ capacity to use digital peer support technologies. The Digital Peer Support Certification received support from clinical staff, peer support specialists, and organizations as well as financial support from funders. As such, implementation of the Digital Peer Support Certification supported adoption of digital peer support technology and flexibility in uptake by peer support specialists. The improvements in the peer support specialists’ capacity were likely due to a combination of the following attributes of the Digital Peer Support Certification: non–time-dependent team learning; nonaversive feedback; inclusion of peer support specialist practice standards; and reasonable accommodations for support. Future studies can build on the *Digital Peer Support Certification* success through employing these components. Next, we will discuss each component in detail.

#### Team Learning

Team learning within an organization is a key mechanism in promoting uptake of new technologies and new practices [23,24]. Team learning is defined as the collective effort of individuals to achieve a common goal [25]. In the learning organization context, team members commonly ask questions, share knowledge, and complement each other's skills [25]. Team learning as part of the Digital Peer Support Certification included printed educational materials paired with group simulation-based training. Research indicates that the impact of printed educational materials on improvements in service delivery is generally small [26]. As such, we combined printed educational materials with simulation-based training. Education paired with simulation-based training offered a risk-free opportunity to practice skills; however, this approach demonstrated only a small change in the peer support specialists’ capacity to use technology. Rather, continuous real-world experience in combination with education and simulation-based training produced the greatest change in capacity, as evidenced by the increase in technology capacity over time. For adult learners, learning occurs through practice in the real world [27]. Our findings indicate that continuous real-world experience may have a greater impact on increasing the capacity to offer digital peer support than education alone paired with simulation-based training.

#### Nonaversive Feedback and Peer Support Practice Standards

Feedback that is perceived as supportive rather than punitive is more likely to positively influence behavior [18,28]. Nonaversive behavioral support is consistent with the values and philosophy of peer support services related to dignity and respect [20]. As such, through supportive feedback, the facilitator (the principal investigator) encouraged peer support specialists to share their experiences and expertise while using the smartphone app and to guide others toward solutions. Peer support practice standards value the experiences and expertise of similar people [12].

#### Reasonable Accommodations

The peer support specialists who participated in the study were offered reasonable accommodations for technology support, which is a service regulated and endorsed by the Americans with Disabilities Act (ADA) [29]. Most employers are obligated to provide reasonable accommodations to a person with a disability (eg, a diagnosis of a serious mental illness) that substantially limits a major life activity or bodily function [29]. According to the ADA, a reasonable accommodation is defined as a “change or adjustment to a job or work environment that permits a qualified applicant or employee with a disability to participate in the job application process, to perform the essential functions of a job, or to enjoy benefits and privileges of employment equal to those enjoyed by employees without disabilities” [30]. For example, training materials are considered to be a type of employment opportunity. As such, Digital Peer Support Certification offers flexible options for support. From ongoing training and professional development to synchronous and asynchronous support services and a 24/7 help desk, this program aims to provide a broad range of reasonable accommodations.

### Limitations of the Study

This study is not without limitations. First, not all peer support specialists attended the audit and feedback sessions. Second, the small sample of peer support specialists may limit the generalizability of the results. Further, in this sample, all peer support specialists owned and used technology prior to using PeerTECH. Thus, all peer support specialists possessed a baseline level of technology capacity, which is consistent with the scientific literature [31]. However, 7 service users borrowed a smartphone; thus, these users had lower initial technology capacity. Low initial technology adoption may have impacted the service users’ rates of technology use. Stratified sampling by technology adoption in future studies may address this potential limitation. Finally, it is not known which learning mechanism produced the greatest effect: the education and simulation training session, the synchronous and asynchronous support services, or the audit and feedback. Future research should control for a time and examine the effects of individual and interactive learning mechanisms to optimize mastery of technology skills by peer support specialists.

### Conclusions

The Digital Peer Support Certification may be an initial step to standardized telehealth training and competencies in the delivery of digital peer support. As people shelter in place and practice social distancing due to COVID-19, a peer support specialist workforce with proper training may play a powerful role in digitally supporting the needs of people in the community. Although the field of digital peer support is in its infancy [32], the expansion of digital peer support through wide-scale Medicaid reimbursements and standards training will potentially have applications in improving the health and wellness of service users during the COVID-19 pandemic. The Digital Peer Support Certification shows promising evidence of increasing the capacity of peer support specialists to use specific digital peer support technology features (eg, SMS text messaging, ecological momentary assessments on smartphone apps, and goal setting). Our findings also highlighted that this capacity was less likely to change with training alone (ie, education paired with simulation-based training); this finding suggests that a combinational knowledge translation approach that includes training *and* management will be more successful.

## Abbreviations

ADA: Americans with Disabilities Act
COVID-19: coronavirus disease

## References

[1] Fortuna KL, DiMilia PR, Lohman MC, Bruce ML, Zubritsky CD, Halaby MR, Walker RM, Brooks JM, Bartels SJ (2018). Feasibility, Acceptability, and Preliminary Effectiveness of a Peer-Delivered and Technology Supported Self-Management Intervention for Older Adults with Serious Mental Illness. Psychiatr Q.

[2] Fortuna KL, Venegas M, Umucu E, Mois G, Walker R, Brooks JM (2019). The Future of Peer Support in Digital Psychiatry: Promise, Progress, and Opportunities. Curr Treat Options Psych.

[3] Fortuna KL, Naslund JA, LaCroix JM, Bianco CL, Brooks JM, Zisman-Ilani Y, Muralidharan A, Deegan P (2020). Digital Peer Support Mental Health Interventions for People With a Lived Experience of a Serious Mental Illness: Systematic Review. JMIR Ment Health.

[4] Fortuna KL, DiMilia PR, Lohman MC, Cotton BP, Cummings JR, Bartels SJ, Batsis JA, Pratt SI (2020). Systematic Review of the Impact of Behavioral Health Homes on Cardiometabolic Risk Factors for Adults With Serious Mental Illness. Psychiatr Serv.

[5] Solomon P (2004). Peer support/peer provided services underlying processes, benefits, and critical ingredients. Psychiatr Rehabil J.

[6] Saeed SA, Johnson TL, Bagga M, Glass O (2017). Training Residents in the Use of Telepsychiatry: Review of the Literature and a Proposed Elective. Psychiatr Q.

[7] Edirippulige S, Armfield N (2016). Education and training to support the use of clinical telehealth: A review of the literature. J Telemed Telecare.

[8] (2014). Telehealth: Specialist video consultations under Medicare. Australian Government Department of Health.

[9] (2016). About Telehealth. The National Telehealth Policy Resource Center.

[10] Fortuna KL, Aschbrenner KA, Lohman MC, Brooks J, Salzer M, Walker R, St George L, Bartels SJ (2018). Smartphone Ownership, Use, and Willingness to Use Smartphones to Provide Peer-Delivered Services: Results from a National Online Survey. Psychiatr Q.

[11] Fortuna K, Barr P, Goldstein C, Walker R, Brewer L, Zagaria A, Bartels S (2019). Application of Community-Engaged Research to Inform the Development and Implementation of a Peer-Delivered Mobile Health Intervention for Adults With Serious Mental Illness. J Particip Med.

[12] (2015). National Practice Guidelines for Peer Supporters. International Assocation of Peer Supporters.

[13] Fortuna KL, Lohman MC, Gill LE, Bruce ML, Bartels SJ (2017). Adapting a Psychosocial Intervention for Smartphone Delivery to Middle-Aged and Older Adults with Serious Mental Illness. Am J Geriatr Psychiatry.

[14] Katz DA, Muehlenbruch DR, Brown RL, Fiore MC, Baker TB, AHRQ Smoking Cessation Guideline Study Group (2004). Effectiveness of implementing the agency for healthcare research and quality smoking cessation clinical practice guideline: a randomized, controlled trial. J Natl Cancer Inst.

[15] Katz DA, Brown RB, Muehlenbruch DR, Fiore MC, Baker TB, AHRQ Smoking Cessation Guideline Study Group (2004). Implementing guidelines for smoking cessation: comparing the efforts of nurses and medical assistants. Am J Prev Med.

[16] Titler M, Herr K, Brooks J, Xie XJ, Ardery G, Schilling ML, Marsh JL, Everett LQ, Clarke WR (2009). Translating research into practice intervention improves management of acute pain in older hip fracture patients. Health Serv Res.

[17] Larson E, Patel S, Evans D, Saiman L (2013). Feedback as a strategy to change behaviour: the devil is in the details. J Eval Clin Pract.

[18] D'Lima DM, Moore J, Bottle A, Brett SJ, Arnold GM, Benn J (2015). Developing effective feedback on quality of anaesthetic care: what are its most valuable characteristics from a clinical perspective?. J Health Serv Res Policy.

[19] Lowe K, Jones E, Horwood S, Gray D, James W, Andrew J, Allen D (2010). The evaluation of periodic service review (PSR) as a practice leadership tool in services for people with intellectual disabilities and challenging behaviour. Tizard Learning Disability Rev.

[20] Evans I, Meyer L, Derer K, Hanashiro R, Evans IM, Meyer LH (1985). An overview of the decision model. An educative approch to behavior problems. A practical decision model for interventions with severely handicapped learners. Vol.

[21] Holtzblatt K, Beyer H (2017). Contextual Design.

[22] (2017). SPSS Statistics for Windows Version 250. IBM.

[23] Edmondson AC (2002). The Local and Variegated Nature of Learning in Organizations: A Group-Level Perspective. Organ Sci.

[24] Edmondson AC, Bohmer RM, Pisano GP (2001). Disrupted Routines: Team Learning and New Technology Implementation in Hospitals. Administrative Science Quarterly.

[25] Wiese CW, Burke CS (2019). Understanding Team Learning Dynamics Over Time. Front Psychol.

[26] Farmer A, Legare F, Turcot L (2008). Printed educational materials: effects on professional practice and health care outcomes. Cochrane Database Syst Rev.

[27] Palis A, Quiros P (2014). Adult learning principles and presentation pearls. Middle East Afr J Ophthalmol.

[28] Larson E, Patel S, Evans DL, Saiman L (2013). Feedback as a strategy to change behaviour: the devil is in the details. J Eval Clin Pract.

[29] The Americans with Disabilities Act of 1990, As Amended. United States Department of Justice, Civil Rights Division.

[30] The ADA: Your Responsibilities as an Employer. US Equal Employment Opportunity Commission.

[31] Fortuna KL, Aschbrenner KA, Lohman MC, Brooks J, Salzer M, Walker R, St George L, Bartels SJ (2018). Smartphone Ownership, Use, and Willingness to Use Smartphones to Provide Peer-Delivered Services: Results from a National Online Survey. Psychiatr Q.

[32] Fortuna KL, Naslund JA, LaCroix JM, Bianco CL, Brooks JM, Zisman-Ilani Y, Muralidharan A, Deegan P (2020). Digital Peer Support Mental Health Interventions for People With a Lived Experience of a Serious Mental Illness: Systematic Review. JMIR Ment Health.

